# Lower limb vascular assessment techniques of podiatrists in the United Kingdom: a national survey

**DOI:** 10.1186/s13047-019-0341-2

**Published:** 2019-05-22

**Authors:** Peta Ellen Tehan, Martin Fox, Sarah Stewart, Susan Matthews, Vivienne Helaine Chuter

**Affiliations:** 1 School of Health Sciences, Faculty of Health and Medicine, University of Newcastle, Ourimbah, NSW 2258 Australia; 2 School of Clinical Sciences, Faculty of Health, Auckland University of Technology, Auckland, New Zealand; 3 Manchester Local Care Organisation, National Health Service, Manchester, UK; 4 Bone and Joint Research Group, Department of Medicine University of Auckland, Auckland, New Zealand

**Keywords:** Non-invasive vascular assessment, Podiatrist, Survey, Doppler, Ankle-brachial index, Toe-brachial index, Toe systolic pressure

## Abstract

**Background:**

Podiatric vascular assessment practices in the United Kingdom (UK) are currently unknown. This study aimed to describe the current practices for performing lower limb vascular assessments by podiatrists in the UK, and, to investigate the effect of practitioner characteristics, including education level and practice setting, on the choice of tests used for these assessments.

**Methods:**

A cross-sectional observational online survey of registered podiatrists in the UK was conducted using SurveyMonkey® between 1st of July and 5th of October 2018. Item content related to: practitioner characteristics, vascular testing methods, barriers to completing vascular assessment, interpretation of vascular assessment techniques, education provision and ongoing management and referral pathways. Descriptive statistics were performed, and multinomial logistic regression analyses were used to determine whether practitioner characteristics could predict the choice of vascular tests used.

**Results:**

Five hundred and eighty five participants accessed the online survey. After drop-outs and exclusions, 307 participants were included in the analyses. Comprehensive vascular assessments had most commonly been performed once (15.8%) or twice (10.4%) in the past week. The most common indicators for performing vascular assessment were symptoms of suspected claudication (89.3%), suspected rest pain (86.0%) and history of diabetes (85.3%). The most common barrier to performing vascular assessment was time constraints (52.4%). Doppler examination (72.3%) was the most frequently reported assessment type, with ankle-brachial index (31.9%) and toe brachial index (5.9%) less frequently performed. There were variable interpretations of vascular test results. The most common topic for education was smoking cessation (69.5%). Most participants (72.2%) were confident in determining ongoing management, with the majority referring to the patient’s general practitioner (67.6%). Practitioner characteristics did not predict the types of vascular tests performed.

**Conclusion:**

The majority of vascular assessments currently performed by podiatrists in the UK are inconsistent with UK or international vascular guidelines and recommendations. Despite this, most podiatrists felt confident in diagnosing, referring and managing patients with peripheral arterial disease (PAD), however many felt they needed more education to feel confident to assist patients with PAD to manage their cardiovascular risk factors.

**Electronic supplementary material:**

The online version of this article (10.1186/s13047-019-0341-2) contains supplementary material, which is available to authorized users.

## Introduction

Peripheral arterial disease (PAD) is estimated to affect > 20 million people worldwide, affects 20% of the population over 60 years of age, and is more common in people with concomitant chronic disease [1–4]. Although PAD can involve arteries throughout the entire body, it most commonly affects those in the lower limb [1]. There is a strong association between PAD and cardiovascular and cerebrovascular disease which may be otherwise undiagnosed and consequently under-managed [5]. It is therefore important to identify the presence of PAD early in the disease process [6] in order to facilitate timely onward referral, manage cardiovascular risk factors and closely monitor the disease process.

Podiatrists potentially play a key role in the early identification of PAD, as they are the main providers of foot health assessment in the community, and typically consult with people who may not demonstrate symptoms of PAD or report themselves to general practitioners [7]. Current international and United Kingdom (UK) national guidelines recommend PAD is tested for using a combination of clinical history taking, pulse palpation, Doppler waveform assessment, ankle-brachial index (ABI), toe systolic pressure and toe-brachial index (TBI) as well as measures of skin perfusion, e.g. transcutaneous oximetry [4, 8–10].

Significant variation in clinical practice and lack of adherence to evidence-based guidelines creates uncertainty in the effectiveness of such testing for identifying those with PAD and achieving any improvement in overall patient outcomes [11]. Recent research has demonstrated that the majority of vascular assessments performed by podiatrists in Australia and New Zealand are inconsistent with current guidelines. The types of testing methods used by podiatrists were shown to be influenced by their practice setting (public versus private), with public podiatrists significantly more likely to undertake lower limb blood pressure testing compared to podiatrists in private practice.

The vascular testing methods used by podiatrists in the UK are currently unknown. Furthermore, it is currently unknown if podiatrists in the UK are utilising guidelines to inform their practice, or if their practice is consistent with guidelines. Therefore, the primary aim of this study was to describe current practices of UK podiatrists in performing lower limb vascular assessments. Secondly, the study aimed to determine whether practitioner education level or practice setting (public versus private) could predict the choice of tests used for lower limb vascular assessments.

## Design and methods

This was a cross-sectional observational survey of UK podiatrists. The anonymous online survey was conducted between 1st of July and 5th of October 2018 using software program SurveyMonkey® (SurveyMonkey Inc., San Mateo, California). Recruitment occurred through bulletin and online advertising through professional bodies including the College of Podiatry, Royal College of Physicians and Surgeons (Faculty of Podiatric Medicine) and Foot in Diabetes UK. Online snowball advertising was also completed via Facebook® and Twitter® by the professional bodies and two of the researchers to their professional networks (MF and PT). Potential participants had access to a web link to the online secure survey which included the participant information statement and consent form. Participants were included if they were podiatrists registered with the Health and Care Professionals Council (HCPC) and currently practicing in the UK. Ethics approval was granted by the University of Newcastle Human Research Ethics Committee prior to the survey being disseminated (H-2012-0384). All participants provided informed consent prior to participation by answering yes following the information statement on the first page of the online survey.

The initial concept for the survey design was based on a previous survey of vascular assessments techniques of podiatrists in Australia and New Zealand [7]. Based on feedback from researchers involved in the previous survey, questions were modified and further adjusted for the UK population. The survey was then piloted by six podiatrists and further amendments made as a result of feedback. Item content related to specific tests used in vascular assessment, factors influencing whether an assessment was performed, interpretation of vascular test results, self-perceived concordance of vascular assessment practice with guidelines, equipment type used and equipment availability (Additional file 1). The survey contained a total of 29 items related to: participant demographics (items 1 to 8), vascular testing methods (items 9 to 13), interpretation of vascular results (items 14 to 21), barriers for assessment and practical aspects of assessment (items 22 to 25), and education and ongoing management (items 26 to 29). Nominal polytomous, continuous, dichotomous and open-ended response types were used.

### Data analysis

Survey participants were included in the analysis if they completed both the participant characteristics and vascular assessment sections of the online survey. All open-ended responses were quantitatively categorised for the purpose of data analysis. Participant characteristics and vascular assessment characteristics were described as n (%) for categorical data or mean (SD) for continuous data. As some participants did not answer all questions, the overall percentages for each question were reported as a percentage of the number of participants who provided responses.

Multinomial logistic regression analyses were undertaken to determine whether practitioner characteristics (education level, or practice setting) could predict the types of tests utilised during lower limb vascular assessments. For the purpose of this analysis, the types of vascular assessment tests were also grouped into categories: observations alone, Doppler testing alone, observation and Doppler, or observation, Doppler and pressure testing. The observation, Doppler and pressure category was used as the reference category. Goodness of fit was determined using the Pearson chi-square statistic. A thematic analysis of content from the open-ended question “What, if any, do you feel the role of a podiatrist is, in assisting patients in managing their cardiovascular health?” was also performed by two of the researchers (PT and VC). Following immersion in the data, patterns of meaning were systematically identified and organised across the dataset. Themes were developed and checked, then finally refined. All analyses were undertaken in SPSS v.25 with a significance level of *P* <  0.05.

## Results

### Participant characteristics

A total of 585 participants accessed the online survey. Five were excluded because they did not consent, 13 were excluded because they were not registered podiatrists currently practicing in the UK and 30 were excluded because they did not complete any of the questions. A total of 537 participants completed the participant characteristics section of the survey. Of these, 307 participants went on to complete the vascular assessment section of the survey and were therefore included in the analyses. Characteristics of the 307 participants are detailed in Table 1. Most participants practiced in the National Health Service (68.7%), were living in England (71.3%) and had a bachelor’s degree or graduate entry master’s degree (63.8%). The mean (SD) years practicing was 16.6 (10.3).

**Table 1:** Participant characteristics

N		307
Podiatry setting	NHS	211 (68.7%)
	Private practice	89 (29.0%)
	Research/education	4 (1.3%)
	Other	3 (1.0%)
Current primary caseload	High risk patients	110 (35.8%)
	Low risk routine patients	82 (19.5%)
	Wound care	60 (19.5%)
	Musculoskeletal	28 (9.1%)
	Rheumatology	2 (0.7%)
	Nail surgery patients	0 (0%)
	Paediatric	1 (0.3%)
	Mixed/other	22 (7.2%)
Place of practice	Town	160 (52.1%)
	City	98 (31.9%)
	Rural	49 (16.0%)
Country	England	219 (71.3%)
	Scotland	43 (14.0%)
	Northern Ireland	22 (7.2%)
	Wales	22 (7.2%)
	Other	1 (0.3%)
Education	Diploma	17 (5.5%)
	Bachelor’s degree or graduate entry Master’s degree	196 (63.8%)
	Post graduate coursework	76 (24.8)
	Higher degree by research only	15 (4.9)
Years practicing, mean (SD)		16.6 (10.3)

Values are presented as n (%), unless otherwise indicated. NHS = National Health Service

### Vascular assessment characteristics

Comprehensive vascular assessments (defined in the survey as more than pulse palpation, to avoid leading participants) were most commonly performed once (15.8%) or twice (10.4%) in the past week (Table 2). The most common estimated time taken to perform a vascular assessment was five minutes (27.5%). Most participants performed vascular assessments as part of a routine visit (81.5%). The three most common barriers in performing vascular assessments were time constraints (52.4%), lack of equipment (42.3%) and lack of experience (33.6%). Nineteen percent of participants reported that there were no barriers in performing vascular assessment. Hand-held Doppler without waveform display was the most common piece of equipment available to participants (86.3%) (Table 3). The three most common diagnostic testing procedures used during a vascular assessment were hand-held Doppler (72.3%) pulse palpation (52.1%), and visual assessment of skin and nails (31.9%).

**Table 2:** General vascular assessment characteristics

Number of comprehensive vascular assessments performed and documented in most recent work day	None	26 (4.8%)
	1	85 (15.8%)
	2	56 (10.4%)
	3	42 (7.8%)
	4	43 (8.0%)
	5	14 (2.6%)
	6	14 (2.6%)
	7	4 (0.7%)
	8	9 (1.7%)
	9	4 (0.7%)
	> 10	10 (1.9%)
Estimated time taken to perform a vascular assessment^a^	5 min	78 (27.5%)
	10 min	72 (25.4%)
	15 min	40 (14.1%)
	20 min	34 (12.0%)
	25 min	3 (1.1%)
	30 min	50 (17.6%)
	40 min	2 (0.7%)
	45 min	5 (1.8%)
Vascular assessment booking practices^b^	As part of a routine visit	194 (81.5%)
	As a separate booking	45 (15.9%)
	Dependent on patient and time required for specific assessments	39 (13.8%)
	Other	5 (1.8%)
Barriers in performing a vascular assessment	Time constraints	161 (52.4%)
	Lack of equipment	130 (42.3%)
	Lack of experience	103 (33.6%)
	Lack of post-graduate vascular training	77 (25.1%)
	There are no barriers	59 (19.2%)
	Vascular team not requesting specific vascular assessments	50 (16.3%)
	Lack of managerial support	39 (12.7%)
	No financial incentive	24 (7.8%)
	Lack of interest	6 (2.0%)

Values are presented as n (%). ^a^ answered by 284 (92.5%) of participants; ^b^ answered by 283 (92.2%) of participants

**Table 3:** Vascular assessment prompts and equipment

Reasons/indicators to perform a vascular assessment	Symptoms of claudication	274 (89.3%)
	Rest pain	264 (86.0%)
	Diabetes	262 (85.3%)
	Active wound	261 (84.7%)
	New patient assessment	252 (82.1%)
	History of poor healing	249 (81.1%)
	Assessment for nail surgery eligibility	221 (71.7%)
	Discolouration of skin	210 (68.4%)
	Cold feet	205 (66.8%)
	Referral request	183 (59.6%)
	Night cramps	183 (59.6%)
	Raynaud’s phenomena	176 (57.3%)
	History of cardiovascular disease	175 (57.0%)
	Chilblains	169 (55.0%)
	Active smoking	168 (54.7%)
	Smoking history	165 (53.7%)
	Burning feet	133 (43.3%)
	History of cerebrovascular disease	119 (38.8%)
	Advanced age	110 (35.8%)
	Hypertension	77 (25.1%)
	Widespread anhidrosis	76 (24.8%)
	Dyslipidaemia	67 (21.8%)
	Other	20 (6.5%)
Vascular assessment equipment available in clinic	Hand-held Doppler without visual waveform display	265 (86.3%)
	Blood Pressure Cuff and sphygmomanometer	166 (54.1%)
	Stethoscope	76 (24.8%)
	Hand-held Doppler with visual waveform display	67 (21.8%)
	Toe pressure cuff	50 (16.3%)
	Automated ankle brachial index machine	27 (8.8%)
	Photoplethysmography probe	19 (6.2%)
	TcPO2 unit	17 (5.5%)
	Automated toe pressure unit	12 (3.9%)
	None of the above	6 (2.0%)
	Other	10 (3.3%)
Diagnostic testing used during a vascular assessment	Hand-held Doppler (waveform and/or pulses)	222 (72.3%)
	Pedal pulse palpation	160 (52.1%)
	Visual assessment of skin and/or nails	98 (31.9%)
	Ankle brachial index	98 (31.9%)
	Patient medical history/symptoms	69 (22.5%)
	Capillary refill time	60 (19.5%)
	Temperature gradient	51 (16.6%)
	Buerger’s test	26 (8.5%)
	Toe brachial index	18 (5.9%)
	Edinburgh Claudication Questionnaire	13 (4.2%)
	Toe systolic pressure	9 (2.9%)
	Brachial Blood pressure	7 (2.3%)
	Pole test	5 (1.6%)
	SpO_2_	2 (0.7%)
	TcP0_2_	1 (0.3%)
	Heart rate	1 (0.3%)

Values are presented as n (%). SpO_2_ = Saturation of Peripheral Oxygen; TcPO_2_ = Transcutaneous Partial Pressure of Oxygen

After categorising the testing methods for regression analysis, 9.8% of respondents used observation alone, 16.9% used Doppler alone, 32.2% used observation and Doppler, and 16.9% used observation, Doppler and lower limb blood pressure measurement (ABI, TBI or systolic toe pressure). The remaining participants used either lower limb blood pressure measurement alone (7.2%), observation plus lower limb blood pressure measurement (5.5%) or Doppler plus lower limb blood pressure measurement (5.5%). Observation plus pressure and Doppler plus lower limb blood pressure measurement were excluded from the regression models due to small sample sizes. The final multinomial regression model fitted the data well which was evident by the non-significant Pearson chi-square (*P* = 0.463). The results showed that podiatrists’ education level and their practice setting were not predictors of the types of vascular assessment tests they used (Table 4).

**Table 4:** Predictors of types of lower limbs vascular tests undertaken by podiatrists

		Observation, Doppler and pressure (ref) (*n* = 49)	Observation alone (*n* = 29)			Doppler alone (*n* = 50)			Observation and Doppler (*n* = 97)		
		N (%)	N (%)	OR (95% CI)	***P***	N (%)	OR (95% CI)	***P***	N (%)	OR (95% CI)	***P***
Education level	Bachelor (**ref**)	30 (61.2%)	17 (58.6%)			28 (56.0%)			70 (72.1%)		
	PG/Research	16 (32.6%)	9 (31.0%)	−.10 (.32, 2.54)	0.85	18 (36.0%)	0.19 (0.51, 2.89)	0.65	24 (24.7%)	−0.47 (0.28, 1.37)	0.24
	Diploma	3 (6.2%)	3 (10.4%)	0.63 (0.34, 10.49)	0.47	4 (8.0%)	0.35 (0.29, 6.93)	0.66	3 (3.1%)	−0.83 (0.08, 2.29)	0.33
Podiatry setting	Public (**ref**)	33 (67.3%)	17 (58.6%)			34 (68.0%)			64 (66.0%)		
	Private	16 (32.7%)	12 (41.4%)	0.44 (0.58, 4.14)	0.38	16 (32.0%)	−0.05 (0.40, 2.26)	0.906	33 (34.0%)	0.12 (0.53, 2.39)	0.75

*Ref* reference category, *OR* odds ratio, *CI* confidence interval, *PG* post graduate study. Bolded P indicates significant difference at < 0.05

### Clinical indicators for vascular assessment and equipment

The three most common indicators for podiatrists to perform vascular assessments were symptoms of suspected claudication (89.3%), suspected rest pain (86%) and presence of diabetes (85.3%) (Table 3). Most participants (86.3%) had access to hand-held Dopplers (without visual waveform display) and blood pressure cuffs and sphygmomanometers (54.1%) in their clinics. Toe systolic pressure cuffs were available to a small proportion (16.3%) of participants. Automated equipment such as automated ABI units (8.8%) and automated toe systolic pressure units (3.9%) were less frequently reported as available.

### Diagnostic interpretation of vascular assessment practices

**Fig. 1:**
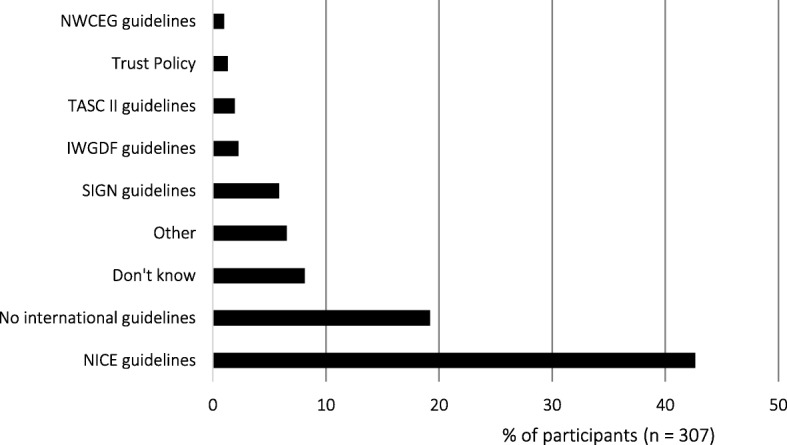
Which international guidelines do you currently utilise to guide your vascular assessment practice?

National Institute for Health and Care Excellence (NICE) guidelines were used to inform vascular assessments by the largest proportion of participants (42.7%), with 19.2% of participants reporting not using any international guidelines (Fig. 1). The most common diagnostic thresholds used for PAD were: < 0.9 for ABI (27.8%), < 50 mmHg for ankle systolic pressure (11.4%), < 0.7 for TBI (5.4%), and <  30 mmHg for toe systolic pressure (4.9%) (Table 5). When using handheld Doppler, the audible output was the most commonly used for interpretation (80.4%). The three most common audible outputs which were considered indicative of PAD were monophasic sounds (82.7%), weak biphasic sounds (21.2%), and quiet/dampened sounds (17.9%).

**Table 5:** Vascular assessment diagnostic interpretation

Ankle brachial pressure index cut-off value for peripheral arterial disease ^a^	< 0.5	14 (4.6%)
	< 0.6	9 (2.9%)
	< 0.7	28 (9.2%)
	< 0.8	39 (12.7%)
	< 0.9	85 (27.8%)
	< 1.0	11 (3.6%)
	< 1.2	1 (0.3%)
	I don’t use ABPI	119 (38.9%)
Absolute ankle pressure cut-off value used for peripheral arterial disease	< 30 mmHg	3 (1.0%)
	< 40 mmHg	5 (1.6%)
	< 50 mmHg	35 (11.4%)
	< 60 mmHg	12 (3.9%)
	< 70 mmHg	12 (3.9%)
	< 80 mmHg	8 (2.6%)
	< 90 mmHg	5 (1.6%)
	< 100 mmHg	7 (2.3%)
	I don’t know	61 (19.9%)
	I do not measure/interpret absolute ankle pressures	159 (51.8%)
Toe brachial pressure index cut-off value used for peripheral arterial disease ^b^	< 0.50	13 (4.4%)
	< 0.55	8 (2.7%)
	< 0.60	7 (2.4%)
	< 0.65	9 (3.0%)
	< 0.70	16 (5.4%)
	< 0.75	2 (0.7%)
	< 0.80	1 (0.3%)
	< 0.90	1 (0.3%)
	< 0.95	1 (0.3%)
	< 1.00	1 (0.3%)
	I don’t use TBPI	236 (80.0%)
Absolute toe pressure cut-off value for Peripheral arterial disease	< 10 mmHg	4 (1.3%)
	< 20 mmHg	3 (1.0%)
	< 30 mmHg	15 (4.9%)
	< 40 mmHg	9 (2.9%)
	< 50 mmHg	8 (2.6%)
	< 60 mmHg	4 (1.3%)
	< 70 mmHg	6 (2.0%)
	< 80 mmHg	2 (0.7%)
	< 90 mmHg	1 (0.3%)
	< 100 mmHg	4 (1.3%)
	I don’t know	27 (8.8%)
	I do not measure absolute toe pressure	226 (73.6%)
Hand-held Doppler interpretation ^a^	Audible output	246 (80.4%)
	Visual output	1 (0.3%)
	Combination of audible and visual output	51 (16.7%)
	I do not use hand-held Doppler	8 (2.6%)
When Audible and visual Doppler outputs are conflicting ^c^	I place more emphasis on visual output	11 (22.0%)
	I place more emphasis on audible output	11 (22.0%)
	I document both outputs separately	23 (46.0%)
	I place less emphasis on Doppler results overall	5 (10.0%)
Doppler Audible output considered indicative of peripheral arterial disease	Monophasic sounds	254 (82.7%)
	Weak biphasic sounds	65 (21.2%)
	Quiet or dampened sounds	55 (17.9%)
	“Whooshing” sounds	41 (13.4%)
	Absent sounds	40 (13.0%)
	Irregular or turbulent sounds	30 (9.8%)
	“Bounding” sounds	20 (6.5%)
	Sluggish or slow sounds	11 (3.6%)
	Sounds which are different between limbs	4 (1.3%)

Values are presented as n (%). ^a^ answered by 306 (99.7%) participants; ^b^ answered by 295 (96.1%) participants; ^c^ answered by 50 (16.3%)

### Education and Management practices

The three most common education topics reportedly discussed with patients following or within their vascular assessments were smoking cessation (69.5%), exercise advice (61.0%) and dietary advice (24.6%) (Table 6). Almost two thirds of podiatrists felt comfortable discussing the association between a vascular event such as heart attack or stroke occurring prematurely due to a diagnosis of PAD (64.4%). Most participants felt comfortable in deciding on the ongoing management of their patients based on vascular assessments (72.29%). General practitioners were the most common referral following a vascular assessment (67.69%).

**Table 6:** Education and management practices following a vascular assessment

Education topics discussed following vascular assessment ^a^	Smoking cessation	189 (69.5%)
	Exercise advice	166 (61.0%)
	Dietary advice	67 (24.6%)
	Diabetes control	59 (21.7%)
	Medication options	55 (20.2%)
	Interpretation of results of assessments	49 (18.0%)
	Lifestyle modifications	46 (16.9%)
	Referral options	42 (15.4%)
	Foot health self-care	41 (15.1%)
	Cardiovascular risk	37 (13.6%)
	Implications of reduced wound healing	35 (12.9%)
	Hypertension management	27 (9.9%)
	Pain management	24 (8.8%)
	Footwear advice	23 (8.5%)
	Weight management	22 (8.1%)
	Cholesterol lowering	19 (7.0%)
	Comorbidities	17 (6.3%)
	Alcohol reduction	11 (4.0%)
	Hosiery advice	9 (3.3%)
	Premature death	9 (3.3%)
	Family history	7 (2.6%)
	Keeping feet warm	6 (2.2%)
	Limb elevation	5 (1.8%)
	Moisturising skin	4 (1.5%)
	Limb compression	2 (0.7%)
	Adequate sleep	2 (0.7%)
	Stress reduction	1 (0.4%)
Comfortable discussing premature vascular event due to PAD diagnosis ^b^	Yes	183 (64.4%)
	No	52 (18.3%)
	Unsure	49 (16.9%)
Comfortable deciding on ongoing management of patient based on vascular assessment ^b^	Yes	205 (72.2%)
	No	30 (10.6%)
	Unsure	49 (17.3%)
Initial referral following vascular assessment ^c^	Vascular surgical team	83 (28.3%)
	General practitioner	158 (67.6%)
	Vascular laboratory	16 (5.5%)
	Podiatry-led PAD team	31 (10.6%)
	Other	14 (4.8%)

Values are presented as n (%). ^a^ 272 (88.6%) of participants; ^b^ 284 (92.5%) of participants; ^c^ 293 (95.4%) of participants. PAD = peripheral arterial disease

### Role of the podiatrist and cardiovascular health

A selection of responses given by participants when asked what they felt the role of a podiatrist was in assisting patients in managing their cardiovascular health is presented in Table 7. Thematic analysis of open-ended response content revealed four themes: that the role of the podiatrist is to educate, empower and encourage patients to manage their own cardiovascular health and risk factors; that more education is needed to enable podiatrists to confidently manage cardiovascular health; that onward referrals and signposting of other services is the podiatrist’s role in cardiovascular management; and, that cardiovascular management is currently out of a podiatrist’s scope of practice.

**Table 7:** Selected responses to: “What, if any, do you feel the role of a podiatrist is, in assisting patients in managing their cardiovascular health?”

*“I feel we are in a privileged position of having more time with patients on a regular basis, having built up a rapport we are able to discuss problems/conditions in a way they feel comfortable which allows better understanding.”*	
*“I think we should be involved, but I need more education.”*	
*“Key role but lacking confidence/ training in addressing difficult conversations with patients”*	
*“Podiatrists can assist in identifying PAD and providing information and education to patients and making appropriate onward referrals”*	
*“There isn’t one - that’s the doctor’s job”*	
*“I don’t feel particularly comfortable with assisting with managing this but I have quite frequently referred the patient back to the GP and highlighted this to them.”*	
*“We are easily accessible for most of the population and i feel we should be more skilled in private practice at assessing vascular problems however due to cost of equipment I feel it will never happen.”*	
*“I think we need to do more in this area, we are in a prime position to be able to identify PAD and have discussions with patients around the cause.”*	
*“I feel all health professionals have a role in assisting patients to manage their cardiovascular health including podiatrists, however I think some health professionals are more qualified to do this than others. As a podiatrist I feel it is slightly out of my scope of practice to manage a patient’s cardiovascular health.”*	
*“Should be to be confident and competent to recognise CV problems and highlight and refer where needed.”*	
*“Important, but overlooked by other health care professionals.”*	
*“I understand it should be paramount in their podiatry experience however feel confidence in this field inhibits my delivery of this advice.”*	
*“It is part of our role but hard when we don’t always have access to information or enough time to assess.”*	

## Discussion

This study describes current practices of UK podiatrists in performing lower limb vascular assessments. Results revealed variable use of assessment techniques, with most podiatrists using subjective methods to assess lower limb vascular status which did not always align with current PAD guidelines, consistent with a previous survey [12]. The current study also demonstrated that practitioner characteristics, including education level and practice setting, did not influence the choice of vascular assessment techniques.

Hand-held Doppler examination of pedal pulses using audible output to interpret the results was the most frequently reported method of assessment. Unlike visual Doppler waveforms, which have demonstrated high sensitivity for identifying PAD [13], audible output has variable reliability when used in podiatry practice [14, 15], thus limiting the extent to which results can be interpreted with confidence. This is most likely related to the type of equipment reported to be available to practitioners in the current study, with most having access to a Doppler with audible output only. The limited access to more reliable vascular testing equipment may impact the effectiveness of current vascular assessment. Although current international guidelines, including the NICE guideline, endorse the use of Doppler assessment for PAD, it is not recommended for use in isolation [4]. This was incongruous with the larger proportion of respondents reporting use of the NICE guideline to inform their vascular assessment practice and may suggest limited implementation of the guideline into clinical practice.

Lower limb blood pressure testing by podiatrists within vascular assessment was limited, with only one third of podiatrists indicating use of the ABI, which was consistent with a shorter average timeframe of five minutes for conducting an assessment. Similarly, there was little reported use of the TBI, toe systolic pressures or transcutaneous oximetry (TCPO2). This is despite the equipment for these tests being accessible to a larger proportion of participants (i.e. photoplythsmography probe). The limited use of TBI and toe systolic pressures may be partially explained by the results of a previous survey, which indicated that podiatrists place less clinical importance on TBI compared to other vascular testing methods, such as Doppler [12]. The limited regular use of lower limb blood pressure testing methods by participants may have contributed to varied interpretation of vascular diagnostic thresholds. Whilst most participants who measured ABI, indicated a value of < 0.9 as indicative of PAD, which is consistent with current international guidelines [16], some participants used lower values which are more reflective of critical limb-threatening ischemia (< 50 mmHg). Toe systolic pressure values were commonly interpreted from the perspective of wound healing capacity rather than identification of PAD, with respondents most frequently choosing the value which is indicative of reduced healing capacity (< 30 mmHg) [17], rather than more recently identified values which may indicate the presence of PAD (< 97 mmHg) [18].

Interestingly, there was no significant influence of practitioners’ education level and practice setting on the types of vascular tests used in a vascular assessment. This may be associated with the high numbers of podiatrists employed in public sector services in the UK, providing a more homogenous practice environment in the study population. Reported barriers for performing vascular assessment were similar to this study [7], including external factors such as time constraints, limited access to equipment and workforce issues including lack of experience, and lack of post-graduate vascular training. Consistent with these findings, thematic analysis of responses to “What, if any, do you feel the role of a podiatrist is, in assisting patients in managing their cardiovascular health?” revealed that many participants wanted further education to be able to confidently practice in this area, and felt it was an area of importance. These factors are in line with published research identifying barriers to implementation of evidence based health care [19].

Our survey findings have identified that greater support is needed to assist UK based podiatrists to implement evidence-based vascular assessment guidelines in clinical practice, consistent with a previous survey [12]. This includes addressing current barriers to performing vascular assessments through work flow- and provider-focused strategies to increase practitioner knowledge and training [19]. In addition, further education is needed to support podiatrists providing more generalised cardiovascular management advice and provide mechanisms to facilitate appropriate referral for effective management. There are current examples of the effectiveness of such changes in improving lower limb vascular assessment and management. These relate to Podiatry-led PAD services emerging in the UK, in which vascular trained podiatrists and vascular nurses are working together to provide local population PAD assessment, diagnosis, triage and management, in partnership with GPs and Vascular teams [20, 21]. These services, whilst small in number, are both clinically and cost effective, having been endorsed as best practice models nationally by NICE and are a successful strategy for improving lower limb vascular management in podiatry practice.

### Potential limitations

This study should be considered in light of some potential limitations. This survey was not validated, therefore may have limited external validity and reproducibility. Our sample size was limited and may not represent podiatrists in the UK who did not respond to the survey invitation. Some participants may have had a higher level of interest in vascular assessment techniques if they were a part of some of the special interest groups where the survey was promoted. The mean years of practice of participants was high, so the results may not be indicative of the practice of more recent graduates. Over-reporting and under-reporting may have been possible, however the use of open-ended response types, and thorough piloting of the survey makes this unlikely.

## Conclusion

The findings of this study demonstrate that podiatrists in the UK rely upon the more subjective vascular assessment tools, such as audible Doppler analysis, clinical observation and pulse palpation to guide their vascular assessment, diagnosis and management plans. Podiatrists however felt confident in diagnosing PAD and guiding further management, despite many not using adequate clinical tests, recommended by current evidence and best practice guidelines. Further podiatric vascular education should focus on this, as well as providing podiatrists with knowledge, skills and confidence to use more objective testing methods and accurately interpret vascular assessment findings, in line with national and international best practice guidelines.

## Additional file

Additional file 1: Copy of survey dissemintaed to participants. (PDF 160 kb)

## References

[1] Shu J, Santulli G (2018). Update on peripheral artery disease: epidemiology and evidence-based facts. Atherosclerosis.

[2] 9. Cardiovascular Disease and Risk Management: <em>Standards of Medical Care in Diabetes—2018</em>. Diabetes Care, 2018. 41(Supplement 1): p. S86-S104. 10.2337/dc18-S009 29222380

[3] Collaboration (2018). P.S.C.a.a.P.C.S., sex-specific relevance of diabetes to occlusive vascular and other mortality: a collaborative meta-analysis of individual data from 980793 adults from 68 prospective studies. The lancet: diabetes and. Endocrinology.

[4] (NICE), N.I.C.E. Lower limb peripheral arterial disease: diagnosis and management (CG147). 2012 August 2012; Available from: http://www.nice.org.uk/guidance/CG147. 26065061

[5] Andras A, F.B., Screening for peripheral arterial disease. Cochrane Database of Systematic Reviews, 2014(4). 10.1002/14651858.CD010835.pub2 PMC11103656 24711093

[6] Smieja M, Hunt D, Edelman D, Etchells E, Comuz J, Simel D (1999). International cooperative Group for Clinical Examination Research. Clinical examination for the detection of protective sensation in the feet of diabetic patients. J Gen Intern Med.

[7] Tehan PE, Chuter VH (2015). Vascular assessment techniques of podiatrists in Australia and New Zealand: a web-based survey. Journal of Foot and Ankle Research.

[8] Norgren L (2007). Inter-society consensus for the Management of Peripheral Arterial Disease (TASC II). J Vasc Surg.

[9] Edmonds M. Best practice guidelines: wound Management in Diabetic Foot Ulcers. Wounds International. 2013.

[10] Hinchliffe R (2016). IWGDF guidance on the diagnosis, prognosis and management of peripheral artery disease in patients with foot ulcers in diabetes. Diabetes Metab Res Rev.

[11] Kennedy PJ, Leathley CM, Hughes CF (2010). Clinical practice variation. Med J Aust.

[12] Normahani P, Mustafa C, Standfield NJ, Duguid C, Fox M, Jaffer U. Management of peripheral arterial disease in diabetes: a national survey of podiatry practice in the United Kingdom. Journal of Foot and Ankle Research. 2018;(29):11. 10.1186/s13047-018-0270-5 PMC5994074 29930710

[13] Tehan P, Sebastian M, Barwick A, Chuter VH (2018). How sensitive and specific is continuous wave Doppler for detecting peripheral arterial disease in people with and without diabetes? A cross-sectional study. Journal of Diabetes and Vascular Disease Research.

[14] Tehan PE, Chuter VH (2015). Use of hand-held Doppler ultrasound examination by podiatrists: a reliability study. J Foot Ankle Res.

[15] Young M, et al. A comparison of the Doppler ultrasound interpretation by student and registered podiatrists. J Foot Ankle Res. 2013;(6):25. 10.1186/1757-1146-6-25 PMC3729664 23849505

[16] Rooke TW (2011). 2011 ACCF/AHA focused update of the guideline for the Management of Patients with Peripheral Artery Disease (updating the 2005 guideline)a report of the American College of Cardiology Foundation/American Heart Association task force on practice guidelines. J Am Coll Cardiol.

[17] Sonter J, Ho A, Chuter VH (2014). The predictive capacity of toe blood pressure and the toe-brachial index for foot wound healing and amputation: a systematic review and meta-analysis. Wound Practice & Research.

[18] Tehan P, Barwick A, Sebastian M, Chuter VH. Diagnostic accuracy of resting systolic toe pressure for diagnosis of peripheral arterial disease in people with and without diabetes: a cross-sectional retrospective case-control study. J Foot Ankle Res. 2017;(10):58. 10.1186/s13047-017-0236-z PMC5735897 29270232

[19] Fischer, F., et al. Barriers and strategies in guideline implementation—a scoping review. in Healthcare. 2016. Multidisciplinary Digital Publishing Institute. 10.3390/healthcare4030036 PMC5041037 27417624

[20] Fox M, Stuart L, Proudman M, Ruff D (2015). A PAD service led by nurses and podiatrists. Nurs Times.

[21] Matthews Sue, Smith Pam, Chadwick Paul, Smyth Vince (2016). Implementing a community-based structured exercise programme for patients with peripheral arterial disease in conjunction with an existing cardiac rehabilitation service results in better outcomes. British Journal of Diabetes.

