# Supplementary material for: Lower limb vascular assessment techniques of podiatrists in the United Kingdom: a national survey
Source: J Foot Ankle Res. 2019 May 22;12:31. doi: 10.1186/s13047-019-0341-2 (PMC6530165; doi:10.1186/s13047-019-0341-2)
Supplement: Supplementary file 1 — Copy of survey dissemintaed to participants. (PDF 160 kb) [file 13047_2019_341_MOESM1_ESM.pdf]

Participant information statement.

Document version 2: 02/03/2018

You are invited to participate in the research project "Vascular assessment techniques among Podiatrists in the United Kingdom" which is being conducted by Mr Martin Fox and Ms Susan Matthews, Vascular Specialist Podiatrists from the Pennine Acute Hospitals NHS Trust, Manchester and Associate Professor Vivienne Chuter and Dr Peta Tehan Discipline of Podiatry at the University of Newcastle, Australia.

Why is the research being done?
The purpose of the research is to ascertain what current practice is among Podiatrists completing vascular assessments in the United Kingdom. This will help determine what future educational training is needed and also if more standardised methods of practice are required in podiatry. The results of this survey may inform the need for promotion of early diagnosis and appropriate management of people with peripheral arterial disease.

Who can participate in the research?
We are seeking Health and Care Professions Council (HCPC) registered Podiatrists in the United Kingdom who are currently practicing.

What choice do you have?
Participation in this research is entirely your choice. Only those people who give their informed consent will be included in the project. Whether or not you decide to participate, your decision will not disadvantage you.

If you do decide to participate, you may withdraw from the project at any time without submitting your survey without giving a reason.

What would you be asked to do?
If you agree to participate, you will be asked to complete the following survey.

How much time will it take?
Participation in this project will take approximately 15 minutes of your time.

What are the risks and benefits of participating?
There are no risks associated with participating in this research.

How will your privacy be protected?
All data will be stored securely at the University of Newcastle by the Principal Researcher and only members of the research team will have access to this data. Data will be retained for at least 5 years. All of your data is unidentifiable. Data will only be saved on electronic file in a coded form which does not identify you in any way. All data will be deleted/destroyed after 5 years. Electronic data will be stored on a password protected computer, paper-based records will be stored in a

locked filling cabinet. Disposal of data will be performed in accordance with university policy (Research Data and Materials Management Procedure document number 000870)

How will the information collected be used?
The results of this study will disseminated via national and international conferences and for papers in scientific journals. Identifying information will not feature in the reporting of this research.

What do you need to do to participate?
Please read this Information Statement and be sure you understand its contents before you consent to participate. If there is anything you do not understand, or you have questions, contact the researcher.

Further information
Thank you in advance for your co-operation with this important effort. Your answers will make a significant contribution to understanding current Podiatry practice in the United Kingdom. If you would like a summary of the survey results, or if you have any questions about this research, please do not hesitate to contact me via email - Peta.Tehan@newcastle.edu.au or Vivienne.Chuter@newcastle.edu.au

Consent

Complaints about this research
This project has been approved by the University's Human Research Ethics Committee, Approval No. H-2012-0384

Should you have concerns about your rights as a participant in this research, or you have a complaint about the manner in which the research is conducted, it may be given to the researcher, or, if an independent person is preferred, to the Human Research Ethics Officer, Research Office, The Chancellery, The University of Newcastle, University Drive, Callaghan NSW 2308, Australia, telephone +61 2 49216333, email Human-Ethics@newcastle.edu.au.

* 1. I have read the participant information statement and am eligible and willing to participate

- [ ] Yes
- [ ] No

* 2. Are you a registered Podiatrist and currently practicing in the United Kingdom?

- [ ] Yes
- [ ] No

Participant Information

* 3. In the past week, the majority of your work has taken place in what kind of Podiatry setting?

- [ ] Private practice
- [ ] NHS
- [ ] Research/education
- [ ] Other (please specify)

* 4. What are the majority of patients currently on your caseload? Choose the most appropriate option

- [ ] High risk patients
- [ ] Wound Care
- [ ] Low risk routine patients
- [ ] Nail surgery patients
- [ ] Musculo-skeletal patients
- [ ] Rheumatology patients
- [ ] Paediatric patients
- [ ] Other (please specify)

* 5. How many years have you been practicing as a Podiatrist?

* 6. Which of the following best describes your primary place of pratice?

- [ ] Cities
- [ ] Towns
- [ ] Rural Locations

* 7. Which country does the majority of your practice take place in?

- [ ] England
- [ ] Northern Ireland
- [ ] Scotland
- [ ] Wales
- [ ] Other (please specify)

* 8. What is the highest level of education you have completed?

- [ ] Diploma
- [ ] Bachelor degree or graduate entry Masters degree
- [ ] Post Graduate Coursework
- [ ] Higher degree by research only

Assessment practices

* 9. In your most recent day of clinical practice, how many comprehensive vascular assessments did you perform and document? (i.e more than pulse palpation)

* 10. Which of the following would prompt you to perform a vascular assessment? You may select multiple boxes

- [ ] Burning feet
- [ ] Active wound
- [ ] Referral request
- [ ] Cold feet
- [ ] Discolouration of skin
- [ ] Widespread anhidrosis
- [ ] Chillblains
- [ ] Night cramps
- [ ] Advanced age
- [ ] Diabetes
- [ ] Hypertension
- [ ] Dyslipidemia
- [ ] Smoking history
- [ ] Active smoking
- [ ] Raynaud's phenomena
- [ ] History of poor healing
- [ ] New patient assessment
- [ ] History of cardiovascular disease
- [ ] History of cerebrovascular disease
- [ ] Assessment for nail surgery eligibility
- [ ] Symptoms of claudication
- [ ] Rest Pain
- [ ] Other (please specify)

* 11. Which of the following vascular assessment equipment do you have access to in your clinic? You may select multiple boxes

- [ ] Stethescope
- [ ] Blood Pressure Cuff and manual Sphygmometer
- [ ] Toe pressure cuff
- [ ] Hand held Doppler without visual waveform display
- [ ] Hand held Doppler with visual waveform display
- [ ] Photoplethysmography probe (PPG)
- [ ] Automated toe pressure unit
- [ ] Automated ankle brachial index machine
- [ ] TcPO2 unit
- [ ] Other (please specify)

* 12. What type of diagnostic testing do you usually use when performing a vascular assessment? i.e. when you need to ascertain the vascular status of the lower limb

* 13. Which, if any, international guidelines do you currently utilise to guide your vascular assessment practice?

* 14. What diagnostic cut-off do you use as an indicator of PAD for the Ankle-Brachial Index?

* 15. What diagnostic cut-off do you use as an indicator of PAD for an systolic/absolute ankle pressure?

16. What diagnostic cut-off do you use an an indicator of PAD for the Toe-brachial index?

* 17. What diagnostic cut-off do you use as an indicator of PAD for a systolic/absolute toe pressure?

18. How do you interpret results from hand-held Doppler?

- ◯ Audio output (sounds)
- ◯ Visual output (waveforms)
- ◯ A combination of audio and visual
- ◯ I do not use Doppler

* 19. When interpreting your Doppler output, what do you do when your visual and auditory signals are conflicting? e.g. monophasic sounds, but biphasic visual waveform or vice versa

- Document whichever is worst
- Document whichever is best
- Document both outputs seperately
- Place more emphasis on audio output
- Place more emphasis on visual output
- Put less emphasis on Doppler result overall
- Other (please specify)

20. What characteristics of Doppler audio output do you consider to be indicative of peripheral arterial disease?

21. What characteristics of Doppler visual output do you consider to be indicative of peripheral arterial disease?

* 22. What is/are the main barriers in performing a vascular assessment in your practice?

- [ ] Time constraints
- [ ] No financial incentive
- [ ] Lack of equipment
- [ ] Lack of interest
- [ ] Lack of experience/confidence in techniques
- [ ] Lack of post-graduate vascular training
- [ ] Lack of managerial support
- [ ] Vascular team not requesting specific vascular assessments
- [ ] There are no barriers
- [ ] Other (please specify)

* 23. How much time do you estimate it takes you to complete a vascular assessment?

○ 5 minutes ○ 10 minutes ○ 15 minutes ○ 20 minutes ○ 25 minutes ○ 30 minutes

Other (please specify)

* 24. Do you book a vascular assessment as a seperate appointment, or is it performed within a routine visit?

○ As part of routine visit

○ As a seperate appointment

○ Other (please specify)

* 25. Which topics do you discuss as part of your education within, or following your vascular assessment?

* 26. Do you feel comfortable discussing with your patients, the risk of premature cardiovascular/cerebrovascular event which is associated with a diagnosis of peripheral arterial disease?

- Yes
- No
- Unsure

* 27. What, if any, do you feel the role of a podiatrist is, in assisting patients in managing their cardiovascular health?

* 28. Do you feel comfortable making a decision regarding ongoing management of a patient, based on the results of your vascular assessment?

- Yes
- No
- Uncertain

29. If you suspect your patient has peripheral arterial disease following your clinical examination, who do you refer to for ongoing management?

- General Practitioner
- Vascular Laboratory
- Vascular surgical team
- Podiatry-led PAD clinic
- Other (please specify)
